# *GSTP1* rs1695 is associated with both hematological toxicity and prognosis of ovarian cancer treated with paclitaxel plus carboplatin combination chemotherapy: a comprehensive analysis using targeted resequencing of 100 pharmacogenes

**DOI:** 10.18632/oncotarget.25712

**Published:** 2018-07-03

**Authors:** Tomoko Yoshihama, Koya Fukunaga, Akira Hirasawa, Hiroyuki Nomura, Tomoko Akahane, Fumio Kataoka, Wataru Yamagami, Daisuke Aoki, Taisei Mushiroda

**Affiliations:** ^1^ Laboratory for Pharmacogenomics, RIKEN Center for Integrative Medical Sciences, Yokohama, Japan; ^2^ Department of Obstetrics and Gynecology, Keio University School of Medicine, Tokyo, Japan

**Keywords:** pharmacogenomics, carboplatin, ovarian cancer, next-generation sequencer, targeted resequencing

## Abstract

**Purpose:**

To find genetic variants that predicted toxicity and/or efficacy of paclitaxel plus carboplatin combination therapy (TC therapy).

**Patients and methods:**

In a retrospective case-control study, we analyzed 320 patients who had received TC therapy for gynecological cancers (ovarian, fallopian tube, peritoneal, uterine, and cervical cancers) and collected their germline DNA. We performed a comprehensive pharmacogenomic analysis using a targeted resequencing panel of 100 pharmacogenes. For 1,013 variants passing QC, case-control association studies and survival analyses were conducted.

**Results:**

*GSTP1* rs1695 showed the smallest p value for hematotoxicity association, and the ^105^Ile wild type allele had a significantly higher risk of severe hematotoxicity (neutropenia G4, thrombocytopenia ≥ G3 and anemia ≥ G3) than the ^105^Val allele (p=0.00034, odds ratio=5.71 (95% confidence interval:1.77-18.44)). Next, we assessed 5-year progression-free survival (PFS) and overall survival (OS) in 56 advanced ovarian cancer patients who received tri-weekly TC as a first-line chemotherapy. Patients with the ^105^Ile/^105^Ile genotype showed significantly better PFS (p=0.00070) and OS (p=0.0012) than those with the ^105^Ile/^105^Val or ^105^Val/^105^Val genotype.

**Conclusion:**

Our study indicates that the *GSTP1* rs1695 ^105^Ile/^105^Ile genotype is associated with both severe hematotoxicity and high efficacy of TC therapy, identifying a possible prognostic indicator for patients with TC therapy.

## INTRODUCTION

Paclitaxel plus carboplatin combination chemotherapy (TC therapy) is an important treatment for gynecological malignancies, including ovarian, peritoneal, fallopian tube, uterine, and cervical cancers. In particular, for ovarian cancer, TC therapy is currently the standard first-line chemotherapy or platinum-sensitive recurrent chemotherapy [1, 2], and carboplatin is considered a key drug in its activity. More recently, a modified administration schedule of TC therapy has been developed, which includes administration of paclitaxel every week (dose-dense TC therapy) [3]. However, the prognosis of patients with ovarian cancer is still poor, especially in the advanced stage [4].

In general, conventional antitumor drugs have a narrow therapeutic window, with a high frequency of adverse drug reactions (ADRs). TC therapy is often associated with serious hematological toxicities, such as neutropenia, thrombocytopenia, and anemia [1, 2]. These toxicities can be a dose-limiting factor, potentially affecting patient quality of life. It is known that the severity or frequency of these adverse events varies between individuals clinically [5]. Therefore, efforts have been made in pharmacogenomic analyses to explore the single-nucleotide polymorphisms (SNPs) associated with toxicity and efficacy of taxanes and platinum agents. Several candidate genes have been reported, such as efflux transporters of the ATP binding cassette (ABC) family, including *ABCB1*, *ABCC2*, and *ABCG2* [6–10]; influx transporters of the solute carrier (SLC) family, including *SLCO1B3* [8, 11]; members of the cytochrome p450 (CYP) family, including *CYP3A4*, *CYP3A5*, and *CYP2C8* [12–15]; genes involved in detoxification, including *GSTP1*, *GSTT1*, and *GSTM1* [16–19]; and genes involved in base-excision DNA repair, including *ERCC1* and *XRCC1* [19–21]. However, to date, no genetic factors have been established as useful biomarkers of toxicity or efficacy of TC therapy.

In our current study, we performed a comprehensive pharmacogenomic analysis by targeted resequencing of 100 pharmacogenes, using a bench top next-generation sequencer. The aim of our study is to identify genetic variants that are associated with the toxicity and efficacy of TC therapy, so that we are able to predict the likelihood of severe toxicity or efficacy in patients before treatment.

## RESULTS

### Patient characteristics

The characteristics of the 320 patients who received TC therapy for gynecological cancers are shown in Table 1. Fifty patients were classified as ADR group and 270 patients were controls. The median age was 54.5 years (range: 35-80) in the ADR group and 54 years (range: 27-80) in the control group. Regarding possible confounding factors, including age, tumor stage by FIGO classification, regimen, and total number of cycles in the observed TC treatment period, we found no significant differences between the ADR group and control groups (Table 1).

**Table 1:** Patient characteristics of the 320 patients

		No. of patients		P value
		ADR group (n=50)	Control group (n=270)	
Age, years	Median	54.5	54	0.093
	Range	35-80	27-80	
Cancer	Ovary	27 (54%)	166 (61%)	
	Uterus	17 (34%)	80 (30%)	
	Cervix	2 (4%)	8 (3%)	
	Ovary+uterus	4 (8%)	13 (5%)	
	Ovary+cervix	0 (0%)	3 (1%)	
Stage	I	18 (36%)	97 (36%)	0.54
	II	6 (12%)	19 (7%)	(I+II vs III+IV)
	III	21 (42%)	114 (42%)	
	IV	5 (10%)	40 (15%)	
Regimen	Dose-dense	12 (24%)	55 (20%)	0.57
	Tri-weekly	38 (76%)	215 (80%)	
Total number of cycles	1	0 (0%)	5 (2%)	0.86
	2	0 (0%)	10 (4%)	
	3	3 (6%)	9 (3%)	
	4	3 (6%)	7 (3%)	
	5	6 (12%)	12 (4%)	
	6	35 (70%)	204 (76%)	
	7	0 (0%)	2 (1%)	
	8	2 (4%)	13 (5%)	
	9	1 (2%)	8 (3%)	
	Median	6	6	
Treatment	1st-line	45 (90%)	253 (94%)	
	Recurrent	5 (10%)	17 (6%)	

ADR: adverse drug reaction

### Targeted resequencing

By the targeted resequencing of 320 subjects, we obtained an output of 35.8 Gbp of sequence data. After a data mapping to human reference sequence (build hg19), on average, 98.5% of the targeted exonic regions (159,347 bp) were covered by at least 20× depth. An average read depth of the targeted regions was 289×. After a QC filtering based on depth < 20× and allele balance < 20%, 1,029 variants were identified. After filtering variants of minor allele frequencies of ≥ 0.001, missing genotype rates of < 10% and p-values less than the cutoff value of < 1.0 × 10^-6^ by the Hardy-Weinberg equilibrium test, 1,013 variants consisting of 11 frameshift deletions, 12 frameshift insertions, 5 nonframeshift deletions, 4 nonframeshift insertions, 601 nonsynonymous single nucleotide variants (SNVs), 23 stop-gain SNVs, 1 stop-loss SNV and 356 synonymous SNVs were used for the further analysis.

### Case-control association study of hematological toxicity

**Figure 1:**
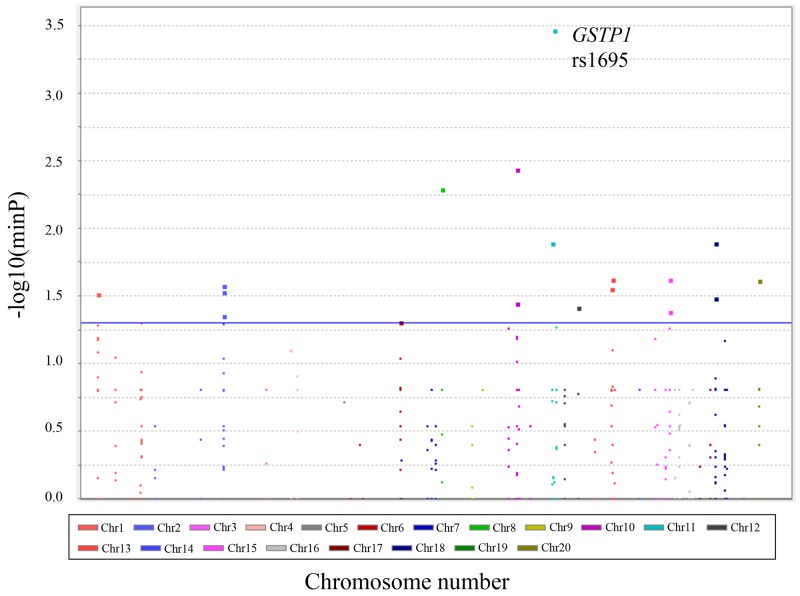
Manhattan plot with hematological toxicities Manhattan plot for case-control association studies of hematological toxicity induced by paclitaxel plus carboplatin combination chemotherapy in Japanese gynecological cancer patients. The –log_10_ (minimal p value) versus chromosome position is plotted for each variant. The blue line indicates the significance level at p=0.05.

The Manhattan plot of the case-control association analyses is shown in Figure 1, and 20 nonsynonymous variants, which showed significant association with ADR grouping, are listed in Table 2. *GSTP1* rs1695 (c.A342G, p.Ile105Val) showed the lowest p-value among them. The ^105^Ile allele (wild type) had a significantly higher risk of severe hematotoxicity than the ^105^Val allele did (p=0.00034, odds ratio=5.71 (95%CI: 1.77-18.44)).

**Table 2:** Twenty nonsynonymous variants which showed significant association

						P value			Genotype count		OR and 95%CI								
			Allele	Allele	Risk	Allelic	Recessive	Dominant	ADR group	Control group	Allelic			Recessive			Dominant		
Chr	SNP ID or position	Gene	1	2	allele	(1vs2)	(vs11)	(vs22)	11/12/22	11/12/22	OR	L95	U95	OR	L95	U95	OR	L95	U95
11	rs1695	GSTP1	G	A	2	0.00034	1	0.00052	0/3/47	5/71/194	5.71	1.77	18.44	2.09	0.11	38.43	6.14	1.85	20.31
10	rs117111102	CYP2C18	T	C	1	0.0037	1	0.0036	0/3/47	0/0/270	38.81	1.99	757.20	5.36	0.11	273.09	39.86	2.03	784.22
8	rs1799930	NAT2	A	G	2	0.0051	0.36	0.0059	0/9/41	9/94/167	2.65	1.29	5.41	3.67	0.21	64.06	2.81	1.31	6.02
11	62752140	SLC22A6	TTTGAACA	-	1	0.013	1	0.013	0/3/47	0/1/269	16.67	1.72	161.92	5.36	0.11	273.09	17.17	1.75	168.60
19	rs3093105	CYP4F2	C	A	1	0.39	0.013	1	3/8/39	1/57/212	1.33	0.71	2.48	17.17	1.75	168.60	1.03	0.50	2.14
13	rs2297322	SLC15A1	T	C	2	0.18	0.024	0.87	3/28/19	51/121/98	1.37	0.87	2.14	3.65	1.09	12.19	1.08	0.58	2.00
15	rs756890035	SLC28A1	C	G	1	0.024	1	0.024	0/2/48	0/0/270	27.44	1.31	575.87	5.36	0.11	273.09	27.89	1.32	589.87
22	rs1135823	CYP2D6	A	C	1	0.024	0.16	0.16	1/0/49	0/0/270	27.44	1.31	575.87	16.39	0.66	408.25	16.39	0.66	408.25
2	rs1042597	UGT1A8	C	G	1	0.047	0.027	0.33	15/22/13	43/134/93	1.58	1.03	2.42	2.26	1.14	4.50	1.50	0.76	2.95
13	rs3765534	ABCC4	T	C	2	0.028	1	0.035	0/7/43	5/73/192	2.41	1.08	5.39	2.09	0.11	38.43	2.50	1.08	5.79
2	rs2070959	UGT1A6	G	A	1	0.032	0.32	0.029	4/25/21	13/97/160	1.67	1.05	2.65	1.72	0.54	5.50	2.01	1.09	3.70
1	rs2297810	CYP4B1	A	G	2	0.039	0.48	0.030	4/17/29	35/124/111	1.68	1.04	2.73	1.71	0.58	5.05	1.98	1.07	3.65
19	rs2108622	CYP4F2	T	C	1	0.23	0.033	0.88	9/16/25	21/110/139	1.31	0.83	2.07	2.60	1.11	6.08	1.06	0.58	1.94
10	rs1057910	CYP2C9	C	A	1	0.036	0.16	0.086	1/4/45	0/11/259	3.07	1.11	8.50	16.39	0.66	408.25	2.62	0.87	7.89
12	rs199876753	SLC16A7	A	T	1	0.042	1	0.038	0/8/42	0/17/253	2.68	1.12	6.38	5.36	0.11	273.09	2.83	1.15	6.98
15	rs8187758	SLC28A1	A	C	1	0.041	0.075	0.091	7/24/19	17/114/139	1.62	1.04	2.54	2.42	0.95	6.19	1.73	0.93	3.21
2	rs11692021	UGT1A7	C	T	1	0.055	0.32	0.044	4/24/22	13/96/161	1.61	1.01	2.57	1.72	0.54	5.50	1.88	1.02	3.46
2	rs6759892	UGT1A6	G	T	1	0.061	0.51	0.045	4/25/21	15/99/156	1.57	0.99	2.49	1.48	0.47	4.65	1.89	1.03	3.48
2	rs1105879	UGT1A6	C	A	1	0.061	0.51	0.045	4/25/21	15/99/156	1.57	0.99	2.49	1.48	0.47	4.65	1.89	1.03	3.48
6	160679680	SLC22A2	C	T	1	0.063	1	0.049	0/15/35	0/45/215	1.86	0.99	3.49	5.16	0.10	263.01	2.05	1.03	4.06

^*^Chr: chromosome number, ADR: adverse drug reaction, OR: odds ratio, CI: confidence interval, L95: lower 95% confidence interval, U95: upper 95% confidence interval.

### Survival analysis

**Figure 2:**
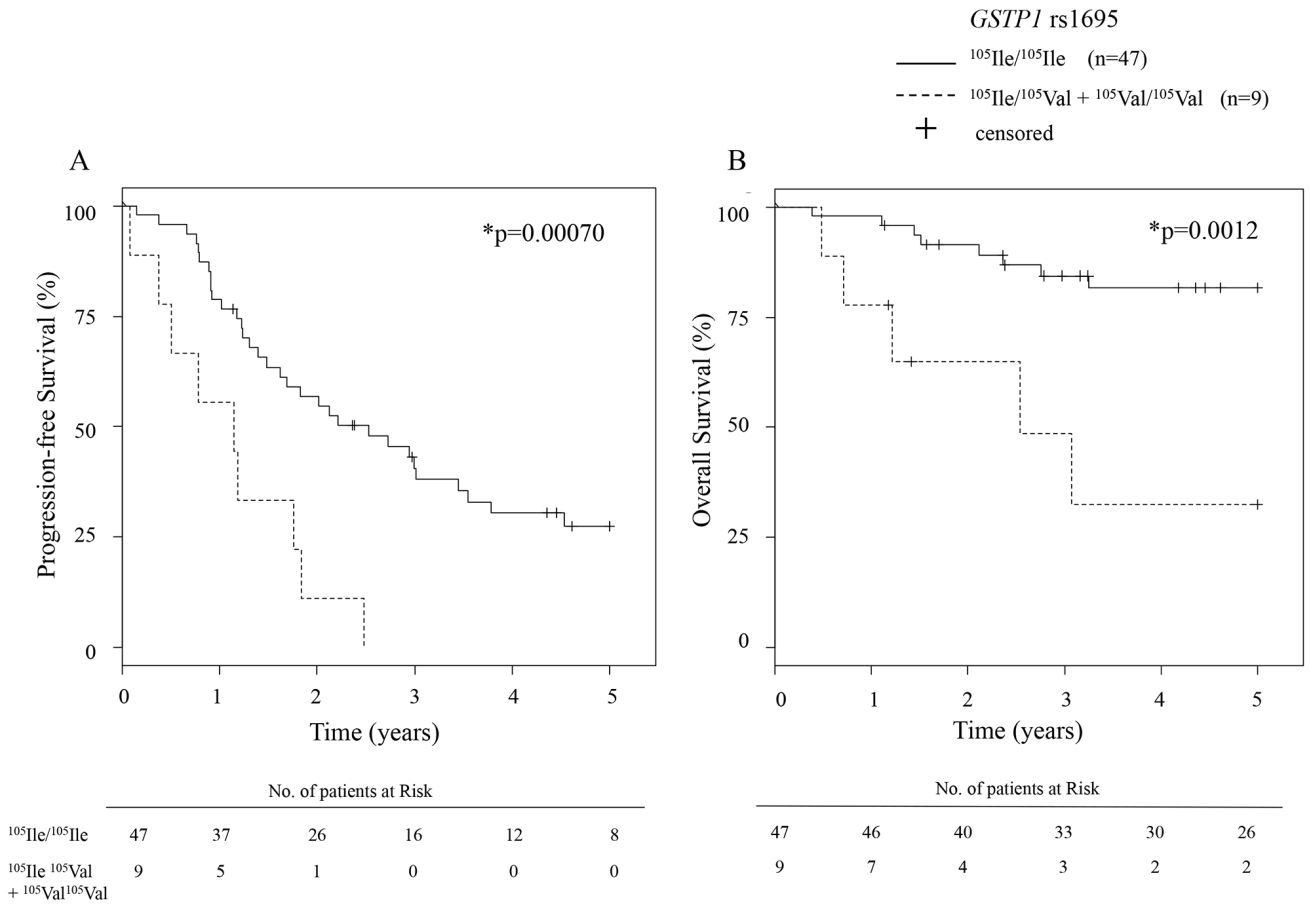
Kaplan-Meier curve for 5-year PFS and OS of tri-weekly TC patients Kaplan-Meier curves of **(A)** progression free survival (PFS) and **(B)** overall survival (OS) of advanced ovarian carcinoma patients who received tri-weekly TC therapy according to *GSTP1* rs1695 genotypes. The risk tables are shown below each plot. TC, paclitaxel plus carboplatin combination.

Next, we compared the 5-year PFS (progression-free survival) and OS (overall survival) of advanced ovarian cancer patients between the *GSTP1* rs1695 genotypes. The characteristics of the 56 patients who received tri-weekly TC therapy as first-line therapy are shown in Table 3, and those of the 55 patients receiving dose-dense TC therapy are shown in Supplementary Table 1. The median follow-up duration was 1764 days (range; 141-5661 days) in the tri-weekly TC group, and 884 days (190-1548 days) in the dose-dense TC group. No patient in the dose-dense TC group reached 5 years of follow-up, because dose-dense TC therapy started in January 2012 in our hospital. The median PFS of tri-weekly TC therapy was 924 days (55-5661 days) in the ^105^Ile/^105^Ile group (n=47) and 417 days (27-905 days) in the ^105^Ile/^105^Val + ^105^Val/^105^Val group (n=9). The median OS of tri-weekly TC therapy was not reached (141-5661 days) in the ^105^Ile/^105^Ile group and was 926 days (177-5077 days) in the ^105^Ile/^105^Val + ^105^Val/^105^Val group. The Kaplan-Meier curves for 5-year PFS and OS of tri-weekly TC therapy are shown in Figure 2. Both 5-year PFS and OS in the ^105^Ile/^105^Ile group were significantly better than those in the ^105^Ile/^105^Val + ^105^Val/^105^Val group were (p=0.00070 and p=0.0012 for PFS and OS, respectively). The PFS and OS with dose-dense TC therapy showed no significant differences between the ^105^Ile/^105^Ile (n=40) and the ^105^Ile/^105^Val + ^105^Val/^105^Val (n=15) groups (p=0.44 and p=0.28 for PFS and OS, respectively, Supplementary Figure 1).

**Table 3:** Patient characteristics of the advanced ovarian cancer patients who received tri-weekly TC therapy

		No. of patients		P value
		^105^Ile/^105^Ile (n=47)	^105^Ile/^105^Val +^105^Val/^105^Val (n=9)	
Age, years	Median	53	51	0.759
	Range	32-73	27-78	
Cancer	Ovary	43 (91%)	9 (100%)	
	Ovary+uterus	4 (9%)	0	
Stage	III	38 (81%)	5 (56%)	0.189
	IV	9 (19%)	4 (44%)	
Debulking status	Complete	22 (47%)	4 (44%)	1.000
	Optimal	17 (36%)	0 (0%)	(complete vs others)
	Suboptimal	8 (17%)	5 (56%)	
Histology	Poorly differentiated	5 (11%)	3 (33%)	
	Serous	23 (49%)	4 (44%)	
	Endometrioid	7 (15%)	0 (0%)	
	Clear	3 (6%)	0 (0%)	
	Others	9 (20%)	2 (22%)	

## DISCUSSION

To date, many researchers have been searching for SNPs associated with susceptibility to taxane- or platinum agent-related toxicities, but, unfortunately, most results have not been replicated [6–24]. Consequently, there have been no established genetic markers that predict the toxicity or efficacy of these drugs, regardless of their clinical importance in the treatment of various cancers. Our current study has some advantages compared with those of previous studies. First, we only included patients receiving paclitaxel plus carboplatin combination chemotherapy, which means that it is possible to eliminate consideration of effects of other taxanes or platinum agents, such as docetaxel, cisplatin and oxaliplatin. Second, we adopted a method of targeted resequencing of 100 notable pharmacokinetics-related genes using a next-generation sequencer, since candidate gene approaches might miss other significant associations. Third, we recruited 320 Japanese patients with detailed clinical data in our hospital. Some previous studies were performed in small sample size, less than one hundred patients, which might have insufficient statistical power to detect significant association.

**Figure 3:**
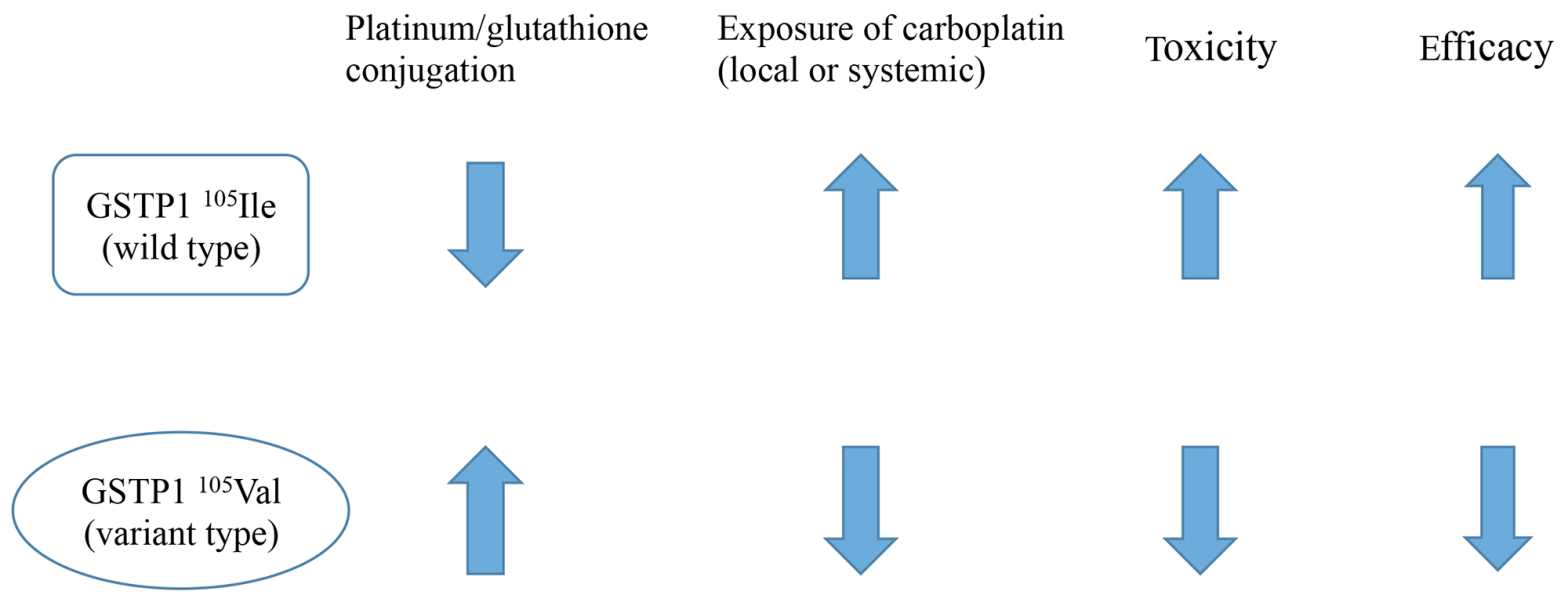
Our model regarding the effect of *GSTP1* rs1695 variants on each pharmacokinetic parameter In our model, the platinum-detoxifying activity of GSTP1 is increased with the rs1695 ^105^Val variant, thus lowering exposure to carboplatin, lowering toxicity, and lowering efficacy.

The case-control association analysis indicated that 20 variants were significantly associated with severe hematological toxicity, with *GSTP1* rs1695 showing the lowest p-value among them (p=0.00034). Glutathione S-transferase P1 (GSTP1) has been shown to metabolize carboplatin *in vitro* [25], and in our results, the ^105^Ile allele was associated with a higher risk of severe hematotoxicity than the ^105^Val allele was (odds ratio=5.71 (95%CI: 1.77-18.44)). We defined severe hematological toxicity as having decreased neutrophil count, platelet count, and hemoglobin concentration. This was based on our assumption that several types of adverse events should be observed simultaneously if they occurred due to the increased drug concentration that was caused by the pharmacogenetic variant. If so, this pharmacokinetic change could also have an influence on the antitumor effect. One of the strongest findings of our study is the association of rs1695 with treatment efficacy, as well as hematological toxicity. Our results indicate that, for advanced ovarian cancers treated with tri-weekly TC, the ^105^Ile/^105^Ile genotype showed significantly better PFS and OS than the ^105^Ile/^105^Val and ^105^Val/^105^Val genotypes did. This result was not observed in the dose-dense TC group, likely because of the shorter observation period and the censoring of many patients. From these results, we were able to develop a model to explain the effects of the genetic variant on the drug efficacy and toxicity (Figure 3). The ^105^Ile/^105^Ile genotype had a lower carboplatin-detoxifying ability, resulting in high carboplatin exposure systematically or locally, which increased both treatment efficacy and risk of hematotoxicity. This model would be of interest to clinicians, especially from a precision-medicine standpoint. If this model is verified, the prognosis of ovarian cancer patients with *GSTP1* ^105^Ile/^105^Val or ^105^Val/^105^Val genotypes could be improved by simply adjusting the carboplatin dose-intensity to optimal efficacy levels, with appropriate management of hematotoxicity.

Glutathione S-transferases (GSTs) are a multigene family of enzymes that catalyze the conjugation of glutathione (GSH) to a variety of electrophilic xenobiotics, eventually forming a mercapturic acid to be excreted into the urine [26, 27]. GSTP1 is a GST isoenzyme, which plays an important role in detoxifying carcinogens, metabolizing chemotherapeutic agents, and regulating the cell cycle and apoptosis [28, 29]. The *GSTP1* gene locus is known to be polymorphic, and three haplotypes have been identified so far, GSTP1^*^A (^105^Ile^114^Ala), GSTP1^*^B (^105^Val^114^Ala), and GSTP1^*^C (^105^Val^114^Val) [30]. Since the ^114^Val (rs1138272, T allele) frequency in the Japanese population is very low, and no patient had this variant in our population, the effect of ^114^Val could be ignored in the current study.

Many studies have shown that GSTP1 can detoxify cisplatin and carboplatin [25, 31–33], and some investigators have discussed the association between GSTP1 and cisplatin resistance [34–36]. The functional effect of rs1695 has also been well studied [25, 27, 30, 32, 37]. Interestingly, the effect of rs1695, with an amino acid substitution in the substrate-binding site, differed depending on the substrates involved [25]. Ishimoto et al. reported that the cytotoxicities induced by cisplatin and carboplatin toward *GSTP1* ^105^Ile-expressing *Escherichia coli* were higher than those of the ^105^Val mutant [25]. In another study, Peklak-Scott et al. found that the conjugation of cisplatin and GSH catalyzed by *GSTP1* ^105^Ile decreased compared to that of ^105^Val [32]. These previous reports showed that the platinum-detoxifying ability of *GSTP1* ^105^Ile was lower than that of ^105^Val, strongly supporting our model.

However, the association of *GSTP1* rs1695 genotypes with the clinical outcomes of platinum-based chemotherapy is still controversial. Some research groups reported the similar findings to ours [19, 38, 39], while others showed the opposite direction of effects of rs1695 [40, 41]. Kim et al. reported that the *GSTP1* rs1695 ^105^Ile/^105^Ile genotype showed higher rates of grade 3 or 4 hematological toxicity than the ^105^Ile/^105^Val and ^105^Val/^105^Val genotypes did [38]. In their study, 96 out of 118 patients (81.3%) received chemotherapy with paclitaxel and carboplatin, 13 (11.1%) with docetaxel and carboplatin, and 9 (7.6%) with paclitaxel and cisplatin. Similarly, Lambrechts et al. reported the ^105^Ile allele to have a higher risk of colony stimulating factor (CSF) use than the ^105^Val allele did [39]. Their study included 266 (82.6%) patients receiving carboplatin and paclitaxel and 56 (17.4%) patients receiving carboplatin monotherapy. They specified their criteria for CSF use as follows: neutropenia grade 4 together with fever >38°C or neutropenia grade 4 for a minimum of 5 consecutive days. In addition, Khrunin et al. reported similar results of ovarian cancer survival revealing that the ^105^Ile/^105^Ile genotype had a significantly increased PFS compared with that of the ^105^Ile/^105^Val or ^105^Val/^105^Val genotype, although their patients received cisplatin-cyclophosphamide regimen [19]. Although some differences existed, overall, our findings corroborated the results of these studies, thus supporting our model strongly. Moreover, our study provides important insights into the treatment of ovarian cancer with TC therapy because there has been, as far as we know, no report that evaluated association of genetic polymorphisms with both toxicity and efficacy of platinum-based anticancer reagents using clinical information of the same patients. We believe that the present findings shed new light on safer and more appropriate chemotherapy for ovarian cancer.

Our model is interesting because it also may explain the ethnic differences in TC therapy efficacy. In the JGOG3016 study [3], the median PFS and OS of tri-weekly TC therapy in Japanese ovarian cancer patients were 17.2 months and not reached, respectively. However, in the GOG-0218 study [42], the investigators reported the median PFS and OS as 10.3 and 39.3 months, respectively, in a population that was composed of non-Hispanic white (84.2%), Asian (6.6%), non-Hispanic black (4.0%), Hispanic (3.4%), and others (1.9%). The Japanese population seems to be more responsive to TC therapy. Interestingly, ethnic differences were observed in the allele frequency of rs1695 ^105^Val, with the Japanese population showing the lowest frequency (0.101), according to the 1000 Genomes Project (http://www.internationalgenome.org/). The higher frequency of wild-type (^105^Ile/^105^Ile) in Japanese may account for the ethnic differences above. To the best of our knowledge, our study is the first to report the association of TC therapy efficacy with *GSTP1* rs1695 in Japanese patients.

Our study had some limitations. First, it was designed as a retrospective case-control study, and there are inherent biases with this type of study. Second, the sample size was small in the survival analysis because only advanced ovarian cancer patients receiving tri-weekly TC or dose-dense TC therapy as first-line chemotherapy had been included for accurate estimation of prognosis. In order to overcome the limitations, the best way is to conduct a replication study using an independent cohort for confirmation of the associations. Therefore, further prospective analyses, including generating pharmacokinetic data or performing replication studies with larger populations, are required in order to firmly establish our model.

In conclusion, we performed a comprehensive pharmacogenomic analysis, using a targeted resequencing panel of 100 pharmacogenes. Our results reveal that *GSTP1* rs1695, known as a functional variant, is associated with both paclitaxel and carboplatin hematotoxicity, and the efficacy of ovarian cancer treatment. Several prior reports *in vivo* and *in vitro* were consistent with our results. We believe that this clinically interesting association will be verified and understood more precisely in the future.

## MATERIALS AND METHODS

### Patients

Keio Women’s Health Biobank (KWB, Tokyo, Japan) was started in 2006 to reposit frozen tissue, formalin-fixed paraffin-embedded (FFPE) tissue, germline DNA, serum, and ascites samples from gynecological patients. This biobank project was approved by the ethics committee of the School of Medicine, Keio University, and written informed consent was obtained from all patients before enrollment.

We found 320 adult female patients, with germline DNA samples available in the KWB, who received at least one cycle of TC therapy for ovarian, fallopian tube, peritoneal, uterine, or cervical cancer from January 1999 to August 2016. Patients who had uncontrolled serious complications were excluded. Clinical data about patient characteristics, ADR information, and data related to survival (i.e., date of recurrence, death, and last visit to the hospital) were collected from the medical records retrospectively. Disease had been confirmed by histologic examination, and the tumor stage had been assessed according to the International Federation of Gynecology and Obstetrics (FIGO) classification.

### Chemotherapy regimens

All patients had received either tri-weekly TC or dose-dense TC therapy, and those TC therapies were performed as either first-line or recurrent treatments. Patients who had received chemotherapy regimen containing taxanes or platinum agents other than paclitaxel or carboplatin (e.g., combination therapy with docetaxel or cisplatin) were excluded. Patients with monotherapy of paclitaxel or carboplatin were also excluded from this study. Tri-weekly TC therapy included administration of paclitaxel (175-180 mg/m^2^) and carboplatin (AUC 6.0 mg·min/mL) on day 1, which was repeated every 3 weeks. Dose-dense TC therapy, on the contrary, included administration of paclitaxel (80 mg/m^2^) on day 1, 8, and 15, and carboplatin (AUC 6.0 mg·min/mL) on day 1, which was repeated every 3 weeks. Appropriate dose reduction was performed for febrile neutropenia, G4 neutropenia that lasted ≥7 days, G4 thrombocytopenia, and non-hematological toxicities ≥G3. Granulocyte-colony stimulating factor (G-CSF) was applied at the discretion of the attending physician, mostly for the patients with febrile neutropenia or prolonged G4 neutropenia. Red blood cell transfusion was also considered if the patient showed anemia ≥G3 and clinical symptoms. Although the standard treatment period consisted of 6 cycles, it could be discontinued if the patient experienced severe adverse events after an appropriate dose-reduction or postponement, or it could be continued beyond 6 cycles if the attending physician deemed extension beneficial.

### Adverse drug reaction evaluation

Complete blood counts had been routinely performed to assess hematological toxicity during TC therapy, and their grades were scored according to the Common Terminology Criteria for Adverse Events, version 4.0. The monitoring period for ADR was from the first administration day to 6 weeks after the final administration of either paclitaxel or carboplatin. The worst grade for neutropenia, thrombocytopenia, and anemia during the treatment period was recorded. We divided the patients based on the severity of hematotoxicity as follows: patients who developed neutropenia G4, thrombocytopenia ≥ G3, and anemia ≥ G3 in one treatment period were designated the “ADR group”, and the other patients were designated the “Control group”. Patients exposed to several TC therapies (e.g., first-line therapy as adjuvant chemotherapy and second-line therapy for the recurrence) were only required to meet the hematological toxicity criteria indicated above during one therapy regimen to be classified into the “ADR group.”

### DNA extraction

Peripheral blood samples had been obtained from patients and stored at -80°C. Genomic DNA was extracted from peripheral lymphocytes using a commercial kit (QIAamp DNA Blood Mini Kit, Qiagen, Hilden, Germany), following manufacturer’s instructions, and stored at 4°C until analysis.

### Targeted resequencing of 100 pharmacogenes

Targeted resequencing of 100 pharmacogenes, which included 37 transporters, 30 cytochrome P450 (CYP) enzymes, 10 uridine diphosphate UDP-glucuronosyltransferases (UGT), five flavin-containing monooxygenases (FMO), four glutathione S-transferases (GST), four sulfotransferases (SULT), and 10 additional genes was performed (Supplementary Figure 2). Of the 100 pharmacogenes, 88 genes are reported in “Clinical annotations” and/or “Variant annotations” of The Pharmacogenetics and Pharmacogenomics Knowledge Base (https://www.pharmgkb.org/), based on scientific review of papers describing association of the variants with ADRs and/or drug efficacy. We selected the remaining 12 genes (*CYP4F3* [43, 44], *CYP4F8* [43, 45], *CYP4F12* [43, 46, 47], *CYP4Z1* [48], *CYP11A1* [49], *CYP26A1* [50], *NUDT1* [51, 52], *SLC10A1* [53, 54], *SLC22A9* [55, 56], *SLC29A2* [57], *SLC29A3* [58], and *SLC46A1* [59]) because a single or multiple papers demonstrated the phenotypic impact of the variant. We enriched the targeted coding regions using multiplex PCR, and added dual barcodes to the PCR products to distinguish each sample. After purification and quantification of PCR products, the pooled libraries were sequenced using the MiSeq Reagent Kit v2 (Illumina, San Diego, CA, USA) with an output of 2 × 250 bp. Sequence reads were aligned to the human reference genome (hg19) using Burrows-Wheeler Aligner (ver. 0.7.4) [60], and then variants were called according to the Genome Analysis Toolkit (GATK, version 3.5.0-g36282e4) [61], using Best Practice Variant Detection recommendations [62].

### Survival analysis

We assessed 5-year PFS and OS as measures of treatment efficacy. For PFS and OS analysis, we focused on advanced ovarian cancer (stage III+IV) from the 320 patients who received tri-weekly (n=56) or dose-dense TC (n=55) therapy as first-line chemotherapy. We analyzed tri-weekly and dose-dense TC separately because previous reports had shown significantly better PFS and OS with dose-dense TC than with tri-weekly TC [3]. PFS was defined as the interval between the first day of TC therapy administration and the day of the first relapse, progression, or death. Relapse and progression were diagnosed by imaging or clinical symptoms. OS was defined as the interval between the first day of TC therapy administration and the day of death.

### Statistical methods

For the 1,013 variants passing QC, case-control association analyses comparing the ADR group with the control group were evaluated by Fisher’s exact test using PLINK version 1.9 [63], considering allelic, dominant, and recessive genetic models. P-values <0.05 were considered to be statistically significant. A Manhattan plot was generated using the minimum p-value among the three genetic models for each variant. Five-year OS and PFS curves were plotted using the Kaplan-Meier method; differences between genotypes were analyzed using the log-rank test. Patients who survived or did not show progression during the observation period were censored at the last confirmation date. The data were analyzed using the freely available statistical software R version 3.3.2. To estimate the differences in patient characteristics between the ADR and control groups, we used a Student’s *t*-test or Fisher’s exact test.

## SUPPLEMENTARY MATERIALS FIGURES AND TABLE



## Abbreviations

TC therapy: paclitaxel plus carboplatin combination therapy
ADRs: adverse drug reactions
GSTP1: Glutathione S-transferase P1
SNPs: single-nucleotide polymorphisms
PFS: progression-free survival
OS: overall survival
