# Supplementary material for: GSTP1 rs1695 is associated with both hematological toxicity and prognosis of ovarian cancer treated with paclitaxel plus carboplatin combination chemotherapy: a comprehensive analysis using targeted resequencing of 100 pharmacogenes
Source: Oncotarget. 2018 Jul 3;9(51):29789–800. doi: 10.18632/oncotarget.25712 (PMC6049855; doi:10.18632/oncotarget.25712)
Supplement: Supplementary file 1 [file oncotarget-09-29789-s001.pdf]

GSTP1 rs1695 is associated with both hematological toxicity and prognosis of ovarian cancer treated with paclitaxel plus carboplatin combination chemotherapy: a comprehensive analysis using targeted resequencing of 100 pharmacogenes

SUPPLEMENTARY MATERIALS

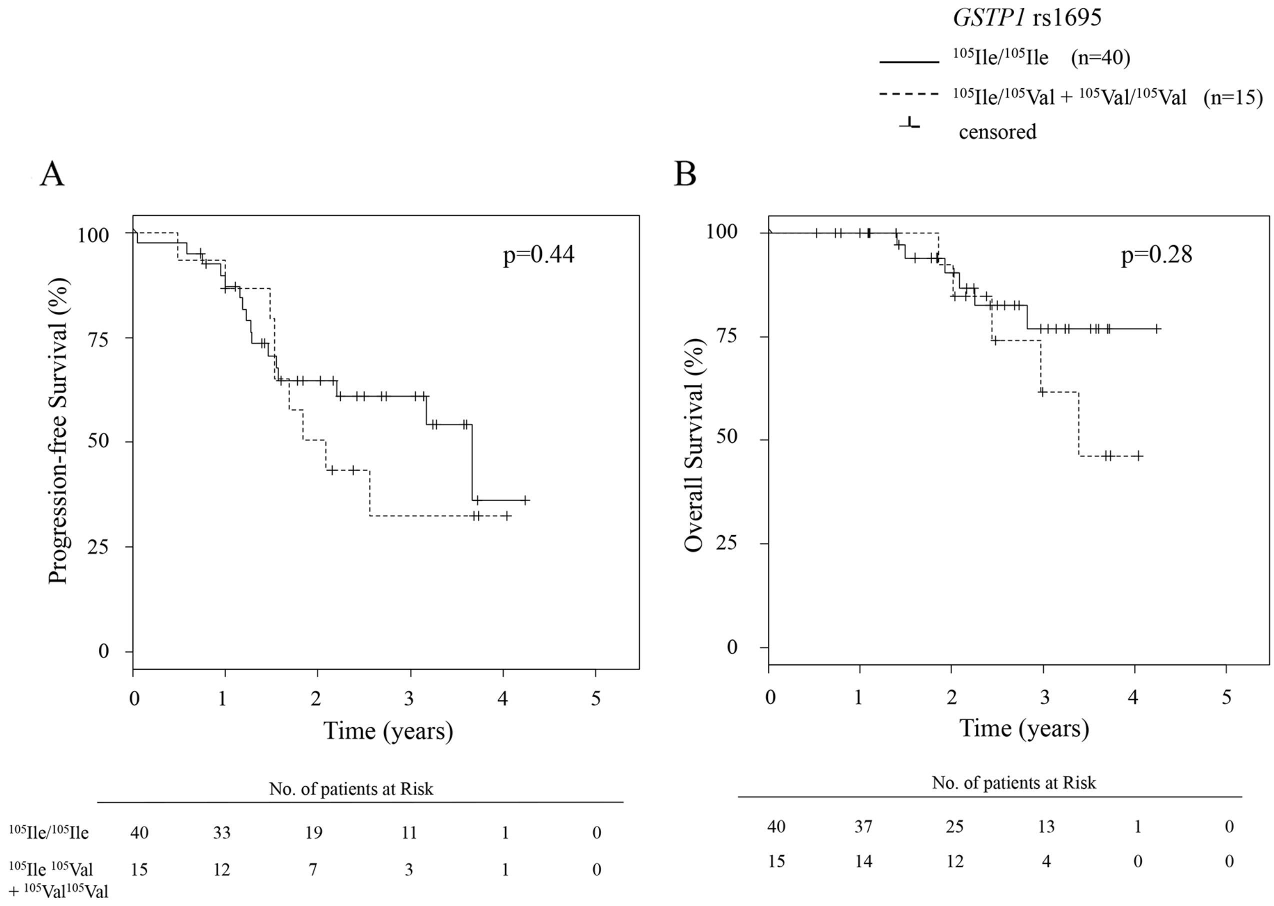

Supplementary Figure 1: Kaplan-Meier curve for PFS and OS of dose-dense TC patients. Kaplan-Meier curves of **(A)** progression free survival (PFS) and **(B)** overall survival (OS) of advanced ovarian carcinoma patients who received dose-dense TC therapy according to *GSTP1* rs1695 genotypes. The risk tables are shown below each plot. TC, paclitaxel plus carboplatin combination.

ABCB1	CYP1A1	CYP2D6	CYP4B1	DPYD	NAT1	SLC19A1	SLC22A12	SLC47A2	UGT1A3
ABCB4	CYP1A2	CYP2E1	CYP4B2	FMO1	NAT2	SLC22A1	SLC28A1	SLCO1B1	UGT1A4
ABCB11	CYP1B1	CYP2J2	CYP4F3	FMO2	NUDT1	SLC22A2	SLC28A2	SLCO1B3	UGT1A5
ABCC1	CYP2A6	CYP2S1	CYP4F8	FMO3	NUDT15	SLC22A3	SLC28A3	SLCO2B1	UGT1A6
ABCC2	CYP2A13	CYP2W1	CYP4F12	FMO4	POR	SLC22A4	SLC29A1	SLUT1A1	UGT1A7
ABCC3	CYP2B6	CYP3A4	CYP4Z1	FMO5	SLC10A1	SLC22A5	SLC29A2	SLUT1A2	UGT1A8
ABCC4	CYP2C8	CYP3A5	CYP11A1	GSTA1	SLC10A2	SLC22A6	SLC29A3	SLUT1E1	UGT1A9
ABCG2	CYP2C9	CYP3A7	CYP17A1	GSTM1	SLC15A1	SLC22A8	SLC31A1	SLUT2B1	UGT1A10
CES1	CYP2C18	CYP3A43	CYP19A1	GSTP1	SLC15A2	SLC22A9	SLC46A1	TPMT	UGT2B7
CES2	CYP2C19	CYP4A11	CYP26A1	GSTT1	SLC16A7	SLC22A11	SLC47A1	UGT1A1	VKORC1

Supplementary Figure 2: Pharmacogenes subjected to targeted resequencing. One-hundred pharmacogenes, including 37 transporters, 30 cytochrome P450 (CYP) enzymes, 10 uridine diphosphate UDP-glucuronosyltransferases (UGT), five flavin-containing monooxygenases (FMO), four glutathione S-transferases (GST), four sulfotransferases (SULT), and 10 other genes were analyzed.

Supplementary Table 1: Patient characteristics of the advanced ovarian cancer patients who received dose-dense TC therapy

		No. of patients		P value
		^{105}Ile/^{105}Ile (n=40)	^{105}Ile/^{105}Val +^{105}Val/^{105}Val (n=15)	
Age, years	Median	55	56	0.449
	Range	34-80	29-71	
Cancer	Ovary	37 (93%)	12 (80%)	
	Ovary+uterus	1 (3%)	3 (20%)	
	Ovary+cervix	2 (5%)	0 (0%)	
Stage	III	29 (73%)	12 (80%)	0.734
	IV	11 (28%)	3 (20%)	
Debulking status	Complete	32 (80%)	8 (53%)	0.086
	Optimal	4 (10%)	6 (40%)	(complete vs others)
	Suboptimal	4 (10%)	1 (7%)	
Histology	Poorly differentiated	0 (0%)	2 (13%)	
	Serous	25 (63%)	8 (53%)	
	Endometrioid	1 (3%)	2 (13%)	
	Clear	4 (10%)	1 (7%)	
	Mucinous	2 (5%)	1 (7%)	
	Others	8 (20%)	1 (7%)	
